# Antiviral Adaptor MAVS Promotes Murine Lupus With a B Cell Autonomous Role

**DOI:** 10.3389/fimmu.2019.02452

**Published:** 2019-10-16

**Authors:** Wenxiang Sun, Hongsheng Wang, Chen-Feng Qi, Juan Wu, Bethany Scott, Silvia Bolland

**Affiliations:** Laboratory of Immunogenetics, National Institute of Allergy and Infectious Diseases, National Institutes of Health, Rockville, MD, United States

**Keywords:** MAVS, FcγRIIB, lupus, antinuclear autoantibody, germinal center, STING, TLR7, type I interferon

## Abstract

Systemic lupus erythematosus (SLE) is an autoimmune disorder characterized by increased production of autoantibodies, which commonly target nuclear antigens, and concomitant deposition of immune complexes that cause inflammation in tissues. SLE is often associated with increased systemic expression of type I interferons, in some cases due to dysregulation in nucleic acid-sensing innate pathways. There is strong genetic evidence for a link between cytoplasmic RNA sensing pathways (RIG-I/MDA5) and SLE, both in human patients and murine models, however questions still remain regarding pathway initiation, cell types involved and downstream effects. Here we show that MAVS, an essential adaptor for RIG-I/MDA5 signaling, is necessary for all symptoms of autoimmune disease that develop spontaneously in the lupus model FcγRIIB^−/−^ mice. This effect was independent of type I interferon signaling, TLR7 expression or STING, all three factors that have been connected to autoimmunity. Mixed bone marrow reconstitution experiments showed reduced occurrence in autoimmune germinal centers and diminished autoantibody production by MAVS-deficient B cells. Thus, MAVS plays a B cell intrinsic role in autoreactive B cell activation that is independent of its anti-viral functions and independent of elevated type I interferon expression.

## Introduction

Dysregulation of a number of innate immune pathways has been associated with the onset and pathogenesis of systemic lupus erythematosus (SLE) (1–3). Studies over the last decade have revealed critical roles for intracellular DNA and RNA sensing pathways in the production of type I interferons (IFNs), a double-edge sword in antiviral defense and autoimmunity (1–3). Among the innate immune pathways that recognize viral RNA and have been linked to SLE are members of the RIG-I-like receptor (RLR) group. This group of RNA sensors includes RIG-I, MDA5 or LGP2 (4–6), which activate the downstream adaptor molecule MAVS (also known as IPS-1, VISA, and Cardif). MAVS undergoes polymerization, described as prion-like filament structures on the outer membrane of mitochondria (7), and recruits numerous adaptor molecules and kinases (8–14). MAVS initiates two well-characterized signaling cascades: 1) the interferon regulatory factors (IRFs) 3 and 7 pathway that leads to the expression of type I/III IFNs and 2) the NF-kB pathway that promotes the expression of proinflammatory proteins (15).

Among pathways upstream of MAVS, multiple genetic studies have linked functional alterations of MDA5 with autoimmune disorders in humans and mice. A missense mutation (G821S) of *Ifih1* (encoding MDA5) results in a constitutively active form of MDA5 that causes mice to develop lupus-like symptoms including glomerulonephritis and a skin rash (16). A similar gain-of-function mutation of R779H in *IFIH1* was reported in a patient with severe early-onset SLE (17). Our previous studies showed that increased copy number of the *Ifih1* gene in mice elevated systemic IFN expression, which was insufficient to induce autoimmune symptoms by itself, but accelerated disease when combined with the lupus prone background of the FcγRIIB deficiency (18).

Genetic studies on the MAVS adaptor itself have identified a loss-of-function mutation of MAVS (C79F) in a subset of SLE patients (19). The mutation was associated with reduced expression of type I IFNs and other inflammatory factors but has not yet proved to have any effect on the pathogenesis of SLE in these patients (19). To better understand the mechanism by which MAVS and related pathways increase SLE susceptibility and to determine the cell types that contribute to this phenotype, we have crossed *Mavs*^−/−^ mice to the autoimmune prone *Fcgr2b*^−/−^ background. We have compared them to *Fcgr2b*^−/−^ mice carrying the STING^gt^ mutation because STING has regulatory roles in development of autoimmune diseases in several models including antigen-induced arthritis (20), autoimmune encephalomyelitis (21), and SLE (22). Deficiency of FcγRIIB in mice is a well-established murine model of SLE and has been shown to induce lupus-like symptoms including increased production of autoantibodies, proteinuria, and glomerulonephritis (23). Mixed bone marrow reconstitution experiments showed that MAVS deficiency abrogated these autoimmune manifestations in a B cell intrinsic manner, which points to a role for MAVS in B cell activation that goes beyond increasing systemic type I IFN expression.

## Materials and Methods

### Mice

*R2*^−/−^ mice were obtained from the Taconic National institute of Allergy and infectious Diseases colony. STING^gt/gt^ and CD45.1 mice were purchased from Jackson Laboratories. *Mavs*^−/−^ mice were kindly provided by Dr. James Z. Chen (15). Mice were bred and housed in a specific pathogen-free facility in the National Institute of Allergy and Infectious Diseases, National institutes of Health. Littermate mice were used. Notes regarding genetic backgrounds: MAVS deficient mice were generated in 129sv background and backcrossed an unknown number of generations to C57BL/6J. FcγRIIB deficient mice were generated in 129sv background and backcrossed 12 generations to C57BL/6N. When these two strains were bred together, the background was a mix of C57BL/6N and 6J. Heterozygous breeders were used to ensure littermates that were MAVS deficient and sufficient. The IgH^a^ allotype was introduced by breeding congenic mice B6.Cg-*Gpi1*^*a*^*Thy1*^*a*^*Igh*^*a*^/J (Jackson laboratory line 1317) to FcγRIIB deficient mice. The *Ifnar1*^−/−^ mice on C57BL/6J background were obtained from the Jackson Laboratory.

Animal studies were approved by the National Institute of Allergy and Infectious Diseases Animal Care and Use Committee.

### TLR7 Stimulation Assay

For *in vivo* stimulation, B6 and *Mavs*^−/−^ mice were injected i.v. with 50 μg of imiquimod (Invivogen) in 100 μl of PBS per animal. Six hours later the serum levels of IL-6 and TNF-α were measured by Mouse ELISA kits (ThermoFisher) according to the manufacturer's instructions. For *in vitro* stimulation, splenic B cells were purified by depletion of CD43^+^ and CD9^+^ cells using an EasySep Mouse PE Positive Selection Kit II (Stemcell Technologies). The purity of cells was determined by flow cytometry to be >97% Supplemental Figure 1. The cells were labeled with 5 μM of CFSE and cultured at 1 × 10^6^/ml with complete RPMI 1640 medium in the presence or absence of 2 μg/ml of imiquimod or various concentrations of R848 for 3 days. Then the cells were stained with 7AAD and analyzed by flow cytometry.

### Flow Cytometry and Antibodies

Spleen, lymph nodes, and BM single cell suspensions were prepared and stained with antibodies listed in Table 1. Biotinylated antibodies were revealed by streptavidin-conjugated fluorochromes. Cells were analyzed with a FACS LSR II or LSRFortessa X-20 (BD Biosciences) and FlowJo software (Treestar).

**Table 1:** List of Antibodies used in FACS and ELISA.

**Antibody**	**Source**	**Application**	**Clone**	**Format**
CD44	Biolegend	FACS	IM7	BV421, Alexa Fluor 700
CD62L	BD	FACS	MEL-14	PE
CD69	Biolegend	FACS	H1.2F3	BV510, PE
CXCR4	BD	FACS	2B11	FITC, BV510, BV605
B220	Biolegend	FACS	RA3-6B2	APC.Cy7, Alexa Fluor 700, Pacific Orange, Pacific blue (PB), Q655
IgM	Biolegend	FACS	RMM-1	PE.Cy7, APC, FITC
IgD	Biolegend	FACS	11-26c.2a	FITC, PB
CD95	BD	FACS	DX2	PE, PE.Cy5
CD138	BD	FACS	281-2	PE, APC
CD19	Biolegend	FACS	6D5	APC.Cy7, Alexa Fluor 700, Pacific Blue, APC
CD11b	BD	FACS	M1/70	PE, BV421, PE.Cy7, BV605
CD11c	eBioscience	FACS	N418	APC, PE,
CD8	eBioscience	FACS	53-6.7	BV510, FITC, APC.Cy7
PNA	Sigma	FACS	NA	Biotin
GL7	BD	FACS	GL7	FITC
CD4	Biolegend	FACS	GK1.5	APC.Cy7, Alexa Fluor 700, Pacific Blue, APC, FITC
PD-1	BD	FACS	29F.1A12	APC
CXCR5	BD	FACS	L138D7	PE, Pacific Blue, PercpCy5.5
CD45.1	Biolegend	FACS	A20	PE.Cy7, Percp-Cy5.5
CD45.2	Biolegend	FACS	104	PE.Cy7, Percp-Cy5.5
IgG2a/c[a]	BD	ELISA	8.3	Biotin
IgG2a/c[b]	BD	ELISA	5.7	Biotin

### BM Chimera

1 × 10^7^ of BM cells of indicated mice were injected i.v. into lethally irradiated mice that received a dose of 940 rad 1 day earlier. Two months later, the reconstituted mice were analyzed for autoantibody production, tissue pathology, and cellular distribution by flow cytometry.

### Proteinuria and ANA Titer Testing

Urinal protein levels were detected with Chemstrip 2GP urine test strips (Roche). Serum ANA titers were determined by the Hep-2 system. Briefly, mice serum samples were diluted at 1:100, 1:300, 1:900, and 1:2,700 with PBS and incubated with Hep-2 substrate slides (MBL, AN-1012) at room temperature for 30 min. After washing twice with PBS for 5 min, the slides were incubated with a secondary anti-mouse IgG-FITC antibody in the dark at room temperature for 30 min. Following washing three times with PBS, the slides were read under a fluorescence microscope and images were taken.

### Histology

Mouse kidney tissues were processed for H&E staining by Histoserv, Inc. (Rockville, MD). The glomerulonephritis scoring was done by measuring the diameters of multiple glomeruli as reported previously (18). Splenic sections were stained with PNA and imaged with an Olympus BX41 microscope (10 × and 40 × objectives) equipped with an Olympus DP71 camera.

### Quantitative Real-Time PCR (qPCR)

Purified B cells were extracted for total RNA using a RNeasy Mini kit (Qiagen). cDNA was synthesized using a Superscript III First-Strand synthesis system (ThermoFisher), followed by PCR using SYBR Green Supermix and primers of *Gapdh* and *Tlr7* (24) in a CFX Connect Real-Time System (Bio-Rad).

### Statistics

Data were analyzed using 2-tailed Student's *t* test (2-tailed) or One-way ANOVA test. *P* < 0.05 is regarded as statistically significant.

## Results

### MAVS Deficiency, but Not the STING^gt^ Mutation, Ameloriates Kidney Pathology, and Increases Survival in FcγRIIB-Deficient Mice

**Figure 1:**
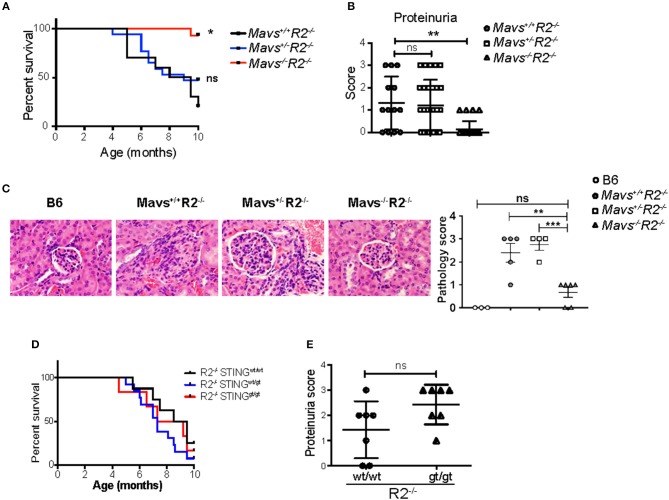
MAVS is required for the autoimmune disease in *R2*^−/−^ mice. **(A)** Survival rates of *Mavs*^+/+^*R2*^−/−^ (*n* = 10), *Mavs*^+/−^*R2*^−/−^ (*n* = 17), and *Mavs*^−/−^*R2*^−/−^ mice (*n* = 14). **(B)** Proteinuria scores of 5-month old mice indicated. Each symbol represents a mouse. **(C)** H&E staining of kidney sections of mice indicated. Magnification, 20×. The right panel is a summary of pathology scores of multiple mice. Each symbol represents a mouse. **(D)** Survival rates of mice of different genotypes indicated. *n* = 6~13. **(E)** Proteinuria scores of 5-month old mice indicated. Each symbol represents a mouse. ^*^p < 0.05; ^**^p < 0.01; ^***^p < 0.001 (One-way ANOVA).

FcγRIIB-deficient mice (*R2*^−/−^) die prematurely due to spontaneous lupus-like autoimmune disease that primarily targets the kidney in the form of lethal glomerulonephritis (23). *R2*^−/−^ mortality starts at 4 months of age, reaching more than 80% penetrance by 10 months of age [(23) and Figure 1A]. To test the role of cytoplasmic nucleic acid sensors in this autoimmune pathology, *R2*^−/−^ mice were crossbred either to MAVS-deficient mice (15), or to mice harboring a gt mutation in the *sting* gene so that it encodes a non-functional STING protein (*STING*^*gt*^) (25). We observed that MAVS-deficient *R2*^−/−^ (*Mavs*^−/−^*R2*^−/−^) mice survived significantly longer than their littermates MAVS-wild type (*Mavs*^+/+^*R2*^−/−^) or MAVS-heterozygous (*Mavs*^+/−^*R2*^−/−^) (Figure 1A). This extended survival rate was associated with markedly reduced proteinuria, a sign of kidney failure, and significantly improved histological scores on kidney pathology in *Mavs*^−/−^*R2*^−/−^ mice compared to *Mavs*^+/+^*R2*^−/−^ or *Mavs*^+/−^*R2*^−/−^ (Figures 1B,C). In contrast, the survival or proteinuria score of *R2*^−/−^ mice was not changed by the presence of the *STING*^*gt*^ mutation (Figures 1D,E).

### MAVS Plays an Essential Role in Promoting Autoreactive Antibodies and Germinal Centers That Goes Beyond the Induction of TLR7 Expression

**Figure 2:**
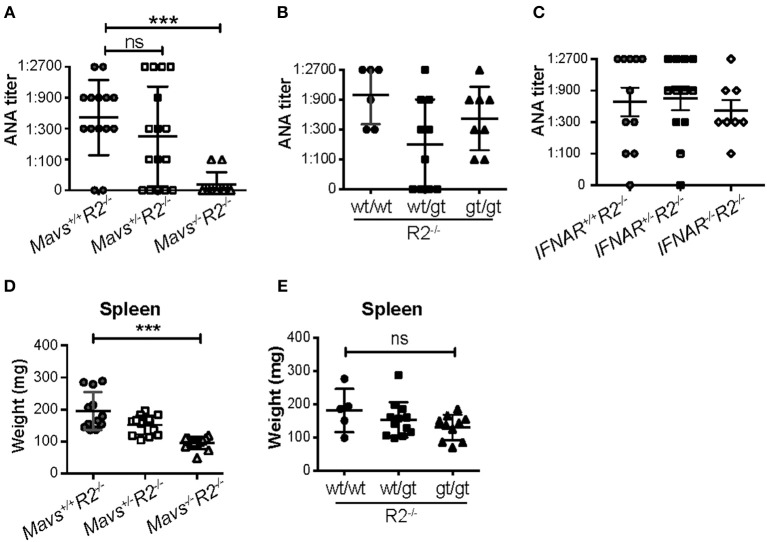
The autoimmune disease in *R2*^−/−^ mice is dependent on MAVS but independent of type I IFNs and STING. **(A–C)** Serum ANA levels of the indicated mice (5-month old) were measured by the Hep2 system. **(D,E)** Spleen weights were measured in 5-month old mice indicated. Each symbol represents a mouse. ^***^p < 0.001 (One-way ANOVA test); ns, not significant.

Serum ANA titers were dramatically reduced in *Mavs*^−/−^*R2*^−/−^ mice compared with MAVS-sufficient *R2*^−/−^ control mice (Figure 2A). In contrast, the *STING*^*gt*^ mutation (heterozygous or homozygous) (Figure 2B) or deficiency in the receptor for type I IFN (Figure 2C) didn't significantly alter ANA titers in *R2*^−/−^ mice. Our results with the IFN receptor deficiency did not depend on the age of the mice and are consistent with those previously reported by Richer et al. (26) using the same mouse model.

**Figure 3:**
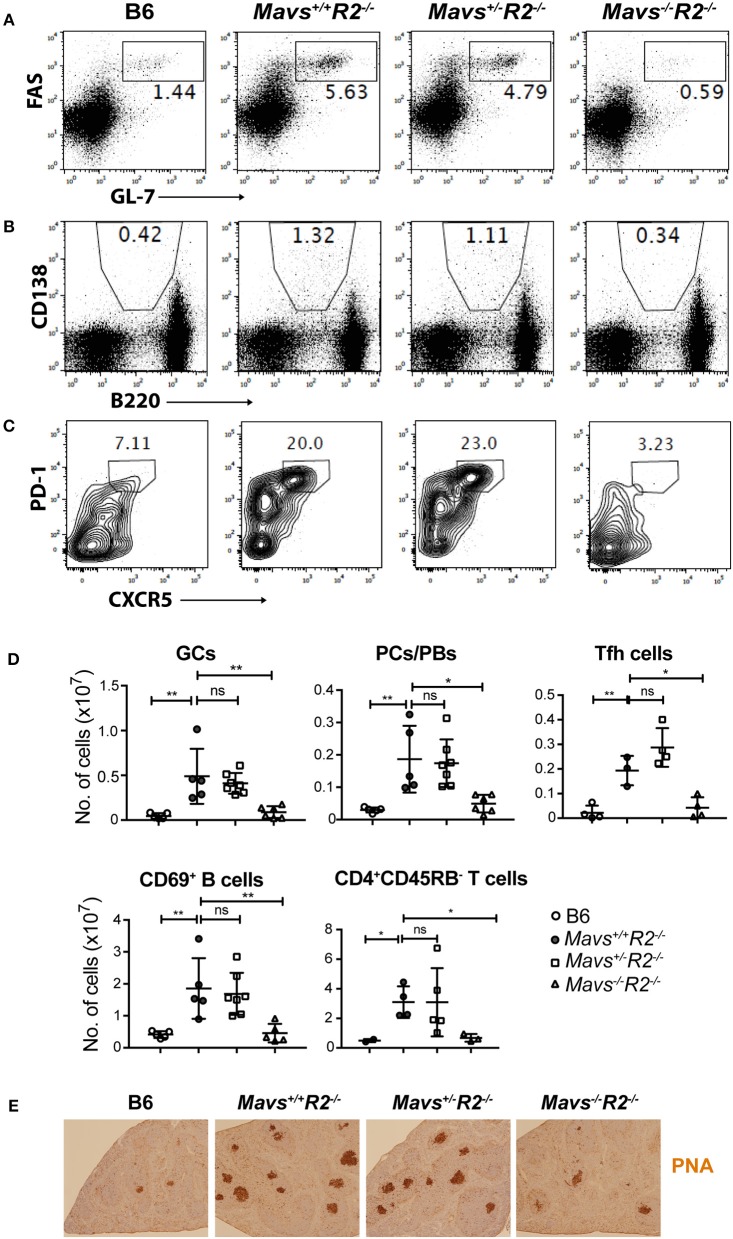
MAVS deficiency prevents spontaneous GCs in *R2*^−/−^ mice. Splenocytes from the indicated mice were stained with antibodies and analyzed by flow cytometry. **(A)** GC B cells (FAS^+^GL-7^+^) were gated on B220^+^ lymphocytes. **(B)** Plasma cells/plasmablasts (PCs/PBs, CD138^+^B220^lo^) were gated on lymphocytes. **(C)** Tfh cells (PD-1^+^CXCR5^+^) were gated on CD4^+^CD62L^−^CD44^+^ cells. The numbers are percentages of cells falling in each gate. **(D)** A summary of multiple mice analyzed as in **A–C**. Each symbol represents a mouse. ^*^p < 0.05; ^**^p < 0.01 (One-way ANOVA). ns, not significant. **(E)** Histological analysis of spleen sections for GCs (stained with PNA, dark brown).

MAVS deficiency, and not the *STING*^*gt*^ mutation, ameloriated the splenomegaly typical of *R2*^−/−^ mice (Figures 2D,E). The range of autoimmune pathology in the various genotypes was also correlated with the extent of immune cell activation. Spleens from *Mavs*^−/−^*R2*^−/−^ mice had reduced numbers of activated B cells (CD69^+^) and activated T cells (CD45RB^−^), as well as reduced number of germinal center B cells, plasma cells/plasmablasts and Tfh cells compared to those in MAVS sufficient control *R2*^−/−^ mice (Figures 3A–D). Immunohistochemical staining of splenic sections dramatically reduced GCs in both the sizes and the numbers (Figure 3E), consistent with the diminished overall pathology score in *Mavs*^−/−^*R2*^−/−^ mice (Figure 1). Thus, we conclude that MAVS deficiency abrogated spontaneous autoimmune GCs and symptoms in autoimmune susceptible *R2*^−/−^ mice.

**Figure 4:**
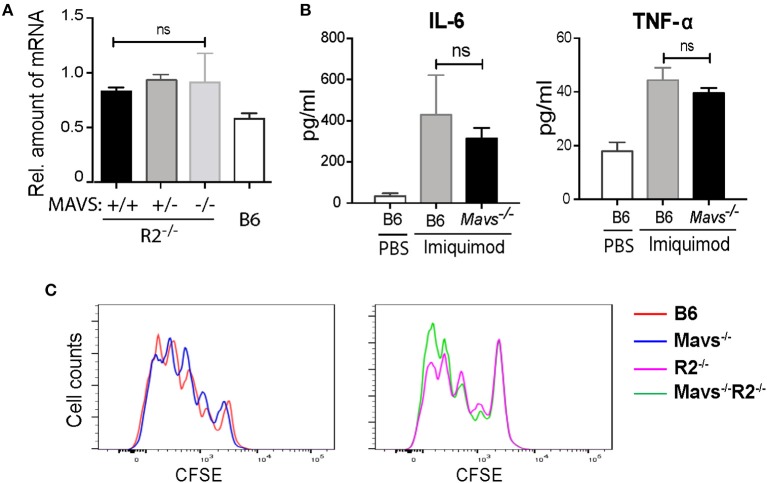
The autoimmune modulatory effect of MAVS in *R2*^−/−^ mice is independent on the TLR7 signaling. **(A)** The expression levels of *Tlr7* mRNA in purified B cells from the indicated mice were determined by qPCR. **(B)** Serum levels of IL-6 and TNF-α detected 6 h after a single injection with imiquimod (50 μg per mouse i.v.). The data are means ± SEM of 3 mice. **(C)** Proliferative response of B cells to imiquimod *in vitro*. Purified B cells (over 97% purity) were labeled with CFSE and cultured with 2 μg/ml of imiquimod for 3 days. Cells were then analyzed by flow cytometry. Data are representative of at least three independent experiments. ns, not significant.

Previous studies using an independent MAVS null strain generated by Xu et al. (also referred to as *Visa*^−/−^) found that the expression of TLR7 was downregulated in the absence of MAVS (24). Interestingly, the expression of TLR7 in the MAVS knockout line generated by Chen's group (15), as used in this study, was not altered (24). We measured TLR7 expression levels in our cohort and found no significant differences between TLR7 transcripts levels in B cells of *Mavs*^−/−^*R2*^−/−^ and control mice (Figure 4A), consistent with previous analyses (24). Injection of the TLR7 ligand imiquimod into mice resulted in production of IL-6 and TNF-α in serum at equivalent levels among different groups of mice, regardless of MAVS expression (Figure 4B). Furthermore, purified B cells from *Mavs*^−/−^*R2*^−/−^ and control mice proliferated at comparable levels following stimulation with imiquimod (Figure 4C) or R848 *in vitro* Supplemental Figure 2. We thus conclude that the expression and function of TLR7 was not affected by MAVS deficiency in the particular murine strain deficient in MAVS that we used for our studies.

### MAVS Promotes Autoimmunity Through Its Function in Bone Marrow-Derived Cells

**Figure 5:**
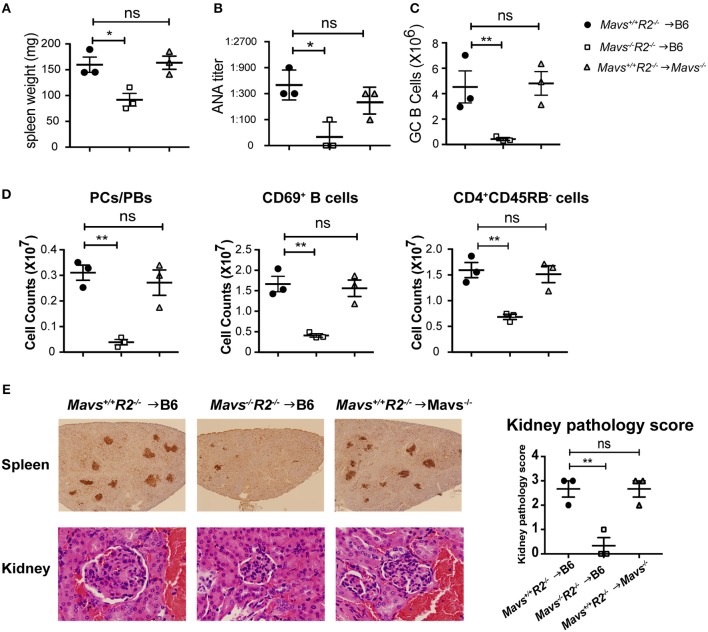
The regulatory effect of MAVS on autoimmunity is bone marrow dependent. **(A–E)** BM chimera mice were generated using donors of *Mavs*^+/+^*R2*^−/−^ or *Mavs*^−/−^*R2*^−/−^ mice and recipients of B6 or *Mavs*^−/−^. Two months after reconstitution, spleen weights **(A)** and serum ANA levels **(B)** of recipients were measured. **(C,D)** Splenic GCs, plasma cells/plasmablasts (PCs/PBs), CD69^+^ B cells, and CD45RB^−^ CD4 T cells were analyzed by flow cytometry. Each symbol represents a mouse. **(E)** Histological analysis of spleen and kidney sections for GCs (top panel, stained with PNA, dark brown) and glomeruli (bottom panel, stained with H&E). The kidney pathology scores of multiple mice are shown in right panel. Each symbol represents a mouse. ^*^p < 0.05; ^**^p < 0.01 (One-way ANOVA test).

To determine the cell types that were responsible for protecting autoimmunity in *Mavs*^−/−^*R2*^−/−^ mice, we generated bone marrow (BM) chimera mice through two different approaches: (i) as a simple BM reconstitution from donor to recipient to test the involvement of bone marrow cells vs. radiation resistant populations, and (ii) a mixed bone marrow reconstitution in which donor cells are comprised of two genotypes in various proportions. For the first approach, we transferred BM cells isolated from *Mavs*^+/+^*R2*^−/−^ or *Mavs*^−/−^*R2*^−/−^ mice into lethally irradiated wild type C57BL/6J (B6) recipient mice. To test the role of MAVS in radiation-resistant populations, we transferred the BM cells of *Mavs*^+/+^*R2*^−/−^ mice into lethally irradiated C57BL/6J. *Mavs*^−/−^ recipients. BM cells of *R2*^−/−^ mice efficiently induced splenomegaly (Figure 5A left) and produced a large amount of serum ANA in B6 recipients (Figure 5B left), similar to that seen in non-manipulated *R2*^−/−^ mice (Figure 2A left). In contrast, BM cells of the *Mavs*^−/−^*R2*^−/−^ origin generated normal sizes of spleens in reconstituted B6 mice (Figure 5A middle) and significantly lower levels of serum ANA (Figure 5B middle). Consistent with this result, the numbers of GC B cells (Figure 5C), plasma cells/plasmablasts, CD69^+^ activated B cells and CD45RB^−^ activated/memory T cells (Figure 5D) of the *R2*^−/−^ origin were higher than those originated from the *Mavs*^−/−^*R2*^−/−^ donor BM. Immunohistochemical staining of spleen sections revealed far fewer and smaller GCs in recipients of the *Mavs*^−/−^*R2*^−/−^ donor BM than those of the *Mavs*^+/+^*R2*^−/−^ donor BM (Figure 5E, top panel). The pathology scores of glomerulonephritis were significantly improved in recipients of *Mavs*^−/−^*R2*^−/−^ BM (Figure 5E, lower and right panels). When testing the effect of MAVS in radiation resistant populations, we observed that MAVS deficiency in recipient mice did not alter the splenomegaly (Figure 5A), autoantibodies (Figure 5B), immune activation (Figures 5C,D) or kidney pathology (Figure 5E right panel) when receiving R2^−/−^ BM. Collectively, these data suggested that BM-derived immune cells were responsible for the autoimmune protective effect in MAVS-deficient *R2*^−/−^ mice.

### MAVS Acts Both in a B Cell Autonomous Manner and in Trans to Regulate Autoreactive GC B Cells

**Figure 6:**
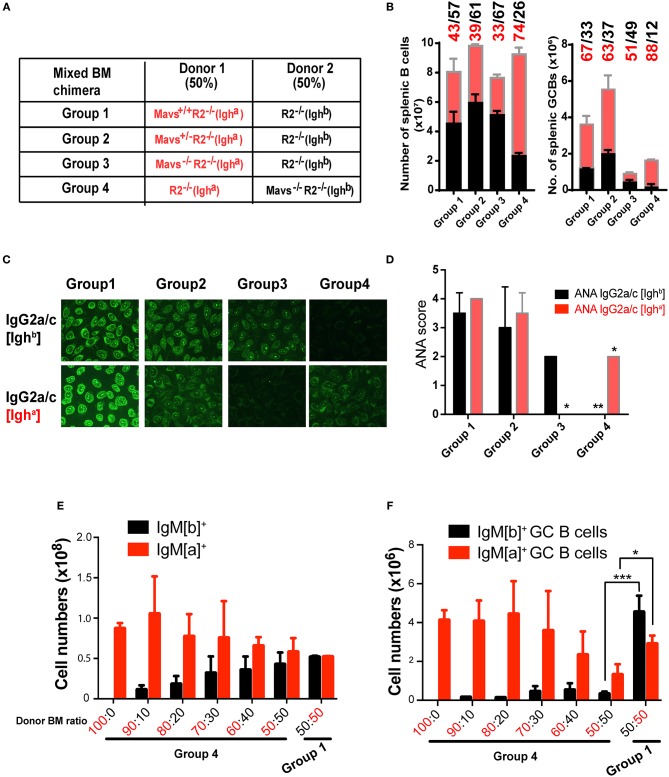
BM chimera mice confirm the B cell intrinsic effect of MAVS on activation and antibody secretion. **(A–D)** Mixed BM chimera mice were generated with donor BM at a 1:1 ratio. **(B)** Two months following reconstitution, the numbers of total B (left panel) and GC B cells (right panel) in recipients were analyzed by flow cytometry. **(C)** The ANA reactivity with an origin of either IgG2a/c [Igh^b^] (top panel) or IgG2a/c[Igh^a^] (bottom panel) was determined by using the Hep2 system. **(D)** A summary of ANA scores of multiple mice. **(E,F)** Donor BM was mixed at the indicated ratio and transferred into recipient mice. The total B cells **(E)** and GC B cells **(F)**of the donor origins were analyzed by flow cytometry and enumerated. Data are means ± SEM of 5–8 mice per group **(A–F)**. ^*^p < 0.05; ^**^p < 0.01; ^***^p < 0.001 (One-way ANOVA test).

For our second approach, we generated mixed BM chimeric mice to examine whether the protective effect of MAVS deficiency was intrinsic to B cells. First we used a mixture containing 50% of *R2*^−/−^ [Igh^b^] BM cells (served as founder cells) and 50% of BM cells from *R2*^−/−^ [Igh^a^] mice that bore one of 3 different MAVS alleles: wild-type (*Mavs*^+/+^, Group 1), heterozygous (*Mavs*^+/−^, Group 2), or null (*Mavs*^−/−^, Group 3) (Figure 6A). As a genetic background control, we reversed the IgH allotype of the donors in Group 4 mice, which received 50% *R2*^−/−^ (Igh^a^) BM and 50% *Mavs*^−/−^*R2*^−/−^ (Igh^b^) BM (Figure 6A). The inclusion of 50% of *R2*^−/−^ BM cells of either Igh^a^ (Group 4) or Igh^b^ (Groups 1–3) background allowed these cells to generate spontaneous autoreactive GCs thereby providing niches for MAVS sufficient or deficient *R2*^−/−^ B cells to function. The production of autoreactive IgG2a/c was analyzed by ELISA using antibodies distinguishing Igh^a^ and Igh^b^ allotypes, allowing us to pinpoint the origin of donor-derived autoantibodies. As shown in Figure 6B, the numbers of splenic B cells with a MAVS-wild type or MAVS-deficient origin were comparable in Group 1 (43% Igh^a^ and 57% Igh^b^). In comparison, the MAVS-null B cells were less represented in both Group 3 and Group 4, independent of allotype: they were 33% of the total in group 3 (Igh^a^ cells) and 26% of the total (Igh^a^ cells) in group 4 (Figure 6B, left panel), compared with 43 and 39% of the total in Group 1 and Group 2, respectively. The numbers of splenic GCs had a slight bias toward the IgH^a^ allotype but were markedly reduced in cells with the *Mavs*^−/−^*R2*^−/−^ origin (Group 3 and 4) compared to those derived from *Mavs*^+/+^*R2*^−/−^ (Group 1) or *Mavs*^+/−^*R2*^−/−^ (Group 2) donors (Figure 6B, right panel). The overall lower numbers of total GCs in Group 3 and Group 4 indicated that the formation of GCs by the founder cells was suppressed by the presence of MAVS deficient cells. This is reminiscent to what Ravetch et al. published when comparing germinal centers generated in bone marrow reconstitution experiments using a mix of *R2*^−/−^ and wild type cells (27). Nevertheless, the significantly lower proportional numbers of GC B cells of *Mavs*^−/−^ (Igh^a^) origin resulted in the total absence of autoreactive IgG2a/c antibodies of the *a* allotype assessed by Hep-2 cell staining (Figure 6C) and serum ANA scores (Figure 6D, group 3 shows ANA score of 0 compared to score of 2 for MAVS-proficient control). The inverted results obtained when the *Mavs*^−/−^ cells express the Igh^a^ allotype (12% of MAVS-deficient cells in Figure 6B; ANA score of 0 in Figure 6D, group 4) rule out any possible effect of genetic background beyond the MAVS deficiency.

We noticed that MAVS deficient B cells had a slight disadvantage in development when tested full B cell numbers in bone marrow reconstitution. As shown in Supplemental Figure 3, *Mavs*^−/−^ mice have smilar numbers of B cell pupulations in bone marrow, but once in the periphery they show a significant disadvantage in follicular cell numbers and an advantage in marginal zone B cell numbers. To get a clear estimate of the effect of MAVS deficiency in autoreactive germinal center B cells independent of this effect on all B cells, we generated mixed bone marrow chimeras with various proportions of donor cells and then measured their numbers after reconstitution in the whole spleen and in germinal centers. In this experiment, B cells expressing the surface IgM^b^ allotype, which in group 4 are MAVS-deficient, have only a slight disantage in development (Figure 6E) but a much more pronounced disadvantage within GC populations (Figure 6F). This effect is also shown in the inverted experiment in group 1, ruling out other genetic effects beyond the MAVS mutation. Collectively, these data suggested that *Mavs*^−/−^*R2*^−/−^ B cells had an intrinsic defect in differentiation into autoreactive GCs and producing IgG2a/c autoantibodies. However we also detected an additional extrinsic effect of MAVS deficiency, either from neighboring B cells acting in trans, or from other immune populations that might affect GC formation.

## Discussion

Our findings reveal a decisive role of MAVS in autoimmune GC responses: the development of autoreactive GCs and autoantibody production in lupus susceptible *R2*^−/−^ mice was nearly completely abrogated by the MAVS deficiency. As a result, the lupus symptoms of *R2*^−/−^ mice including glomerulonephritis and proteinuria were significantly improved and the lifespan of the mice was prolonged. Moreover, elevated expression of TLR7 and the STING signaling were not required in this model. At the cellular level, it is the B cells that were mainly responsible for the protective effect in *Mavs*^−/−^*R2*^−/−^ mice, arguing for a role for MAVS in B cell tolerance that goes beyond systemic expression of type I interferon.

We currently don't have a full mechanistic view on how the MAVS pathway might enable autoreactive B cells to differentiate into GC B cells and autoantibody-secreting cells. Recent findings suggest that MAVS may function through a viral independent mechanism, although none of the published findings point to B cell intrinsic effects. It has been shown that MAVS can undergo oligomerization by reactive oxygen species (ROS) generated by oxidative stress (28). Interestingly, peripheral blood lymphocytes of SLE patients contain spontaneous MAVS oligomerization, which correlates with increased production of type I IFNs. Furthermore, cells expressing the loss-of-function mutation of MAVS (C79F) contain lower levels of ROS-dependent MAVS oligomerization (28). It remains unclear, however, how ROS induces MAVS oligomerization. One possibility is that ROS damages endogenous RNA, especially the ribosomal RNA (29), which leads to recognition by MDA5 and then triggers MAVS oligomerization. It has been shown that accumulation of endogenous RNA caused by dysregulated RNA editing (e.g., adenosine deaminase ADAR1 deficiency) indeed induces virus-independent MAVS activation (30). These findings collectively support an anti-viral independent mechanism for MAVS function in B cell biology that is compatible with our findings of a B cell autonomous effect of MAVS in autoimmunity.

MAVS deficiency diminishes even further the spontaneously induced low background interferon expression levels. This can affect expression of genes that are easily induced by type I interferon, one of them being TLR7, which is well-established as susceptibility factor in lupus development, particulary in the context of the *R2*^−/−^ model and the Yaa autoimmune modifier (31, 32). However, our data points to a TLR7-independent role of MAVS in the development of autoimmunity. Both expression levels and functional testing of TLR7 were not altered in *Mavs*^−/−^ B cells [(24) and Figure 4]. Furthermore, expression levels of TLR7 are tightly linked to type I IFNs, and our data from mice deficient in IFNAR demonstrated that IFN signaling is dispensable for the phenotype. It is worth to note that another strain of MAVS knockout (*VISA*^−/−^) generated by Xu et al. showed a role of MAVS in regulation of TLR7 expression (24). This could be due to differences in genetic backgound, which has increasingly drawn attention from the scientific community (33, 34).

Contrasting the essential role for MAVS in B cell autoimmunity, we found no such role for the cytoplasmic DNA sensor STING, also known to induce type I IFN. The STING^gt^ mutation did not change the survival rate of FcγRIIB deficient mice or altered autoantibody levels (Figure 2). Previous reports addressing the link of the STING pathway with autoimmunity have provided varied views: STING is essential for the phenotype of *Trex1*^−/−^ mice in which accumulation of endogenous DNA substrates triggers production of type I IFNs (35). In contrast, STING has a suppressive effect in the phenotype of lpr mice (22). While these two reports connect STING with autoimmunity, albeit in opposite directions, our data points to a non-involvement for this pathway in our mouse model. One explanation for these differences could be that our model is independent of type I IFN expression (Figure 2C) while the *Trex1*^−/−^ mouse model is completely based on augmented IFN levels (35).

In summary, we have uncovered anti-viral independent roles for MAVS in regulation of autoimmunity. MAVS deficiency correlates with diminished spontaneous autoimmune GCs and autoantibodies in autoimmune susceptible *R2*^−/−^ mice. Our results parallel the findings in SLE patients who bear a loss-of-function mutation of *MAVS* with reduced levels of type I IFNs and autoreactive anti-RNA binding protein antibodies. These findings broaden our understandings of MAVS functions in anti-viral immunity and autoimmunity with important implications for future therapeutic strategies to target the MAVS pathway in the control of SLE.

## Data Availability Statement

All datasets generated for this study are included in the manuscript/Supplementary Files.

## Ethics Statement

The animal study was reviewed and approved by the National Institute of Allergy and Infectious Diseases Animal Care and Use Committee.

## Author Contributions

WS, HW, C-FQ, JW, and BS performed the experiments and analyzed the data. SB conceived and designed the study and analyzed the data. WS, HW, and SB wrote the paper.

### Conflict of Interest

The authors declare that the research was conducted in the absence of any commercial or financial relationships that could be construed as a potential conflict of interest.
