# Supplementary material for: Antiviral Adaptor MAVS Promotes Murine Lupus With a B Cell Autonomous Role
Source: Front Immunol. 2019 Oct 16;10:2452. doi: 10.3389/fimmu.2019.02452 (PMC6805724; doi:10.3389/fimmu.2019.02452)
Supplement: Supplementary file 1 [file Data_Sheet_1.PDF]

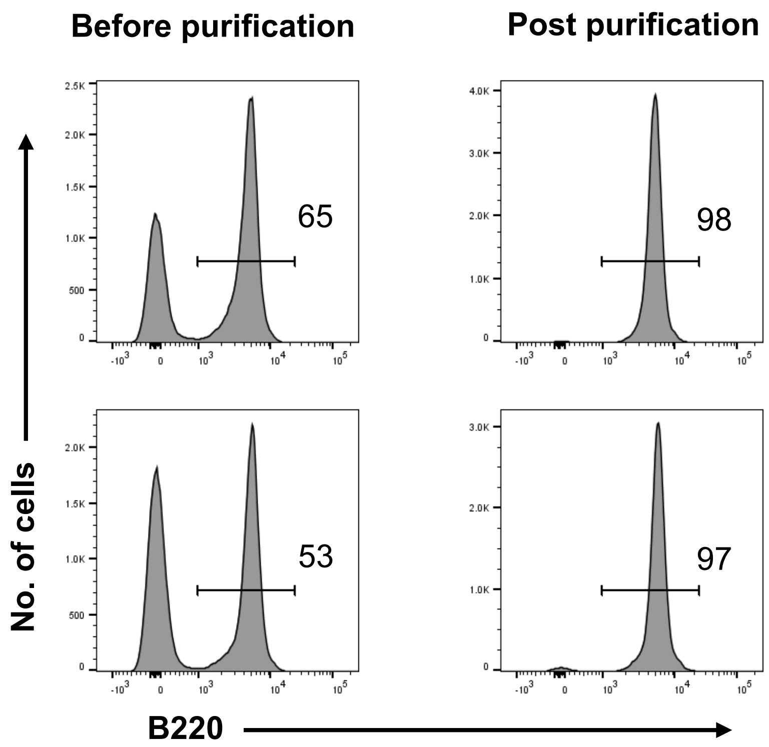


**Supplemental Fig. S1. B cell purity test.** Splenocytes were purified using magnet beads to deplete $CD43^+$ and $CD9^+$ cells according to the manufacturer's instructions. Cells were then stained with anti-B220-Alexa Fluro700 and analyzed by flow cytometry. The cells were gated on $7AAD^-$ viable singlets. The numbers are percentages of cells falling in each gate.

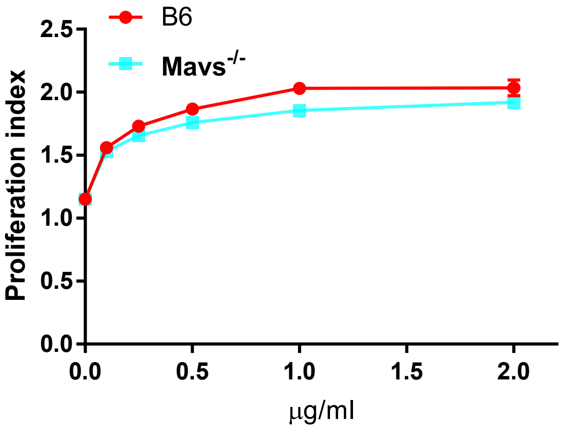


**Supplemental Fig. S2.** Dose-dependent proliferation of B cells to TLR7 ligand stimulation. Splenic B cells purified from B6 and Mavs-deficient mice were labeled with CFSE and stimulated with the indicated concentrations of R848 for 3 days. The cells were then analyzed by flow cytometry. Cells were gated on viable ($7AAD^-$) singlets. The proliferation index was calculated by the FlowJo software. Data are duplicate wells of two mice per group.

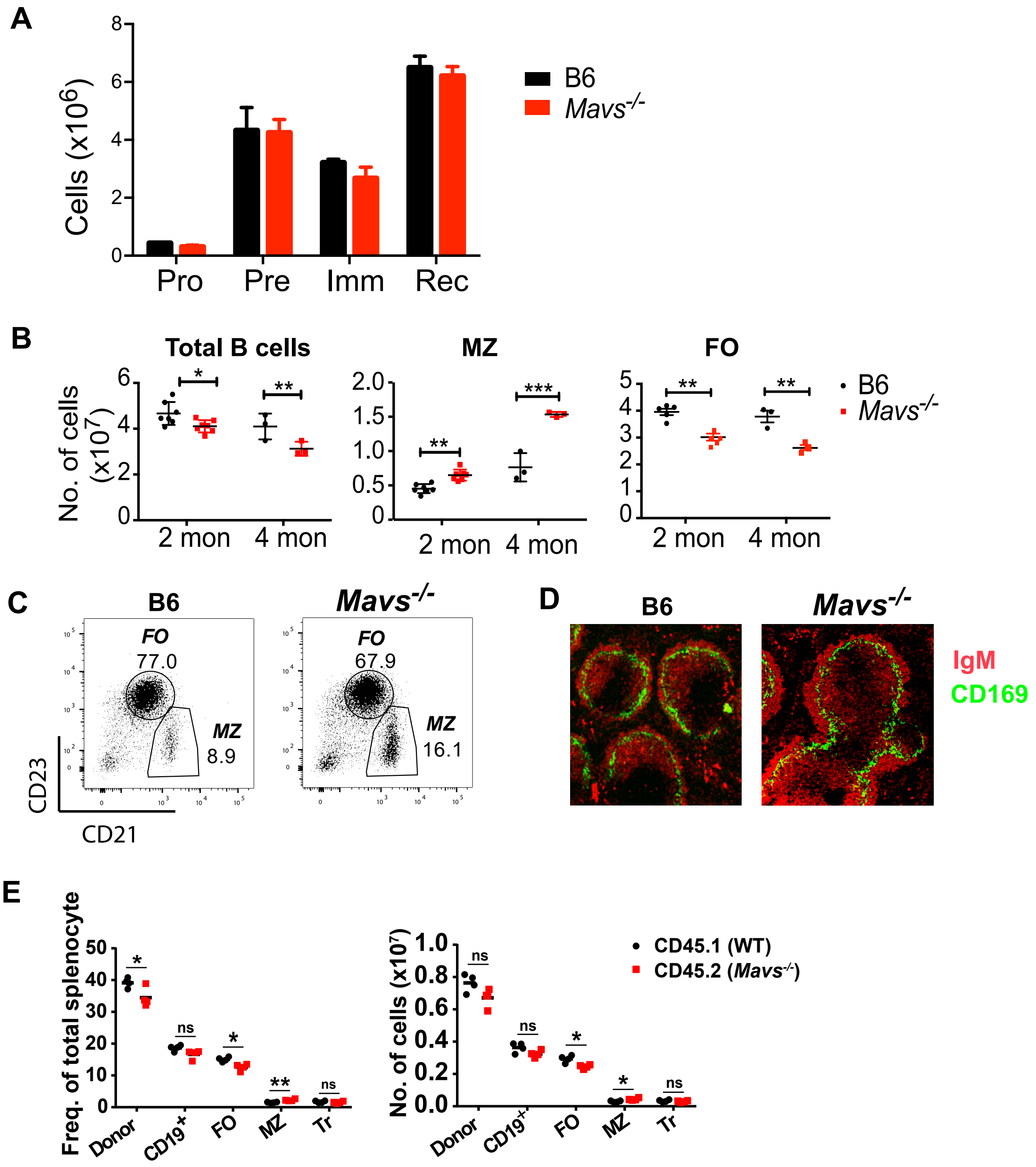


**Supplemental Fig. S3. B cell development in *Mavs*$^{-/-}$ mice.** BM (A) cells were stained with antibodies against B220, CD43, c-kit, CD25, CD19 and IgM and analyzed by flow cytometry. The cells were gated on viable singlets. The definitions of pro-B (Pro), pre-B (Pre), Immature (Imm) and recirculating (Rec) B cells are: B220$^{+}$IgM$^{-}$CD43$^{+}$CD25$^{-}$C-kit$^{+}$CD19$^{+}$, B220$^{+}$IgM$^{-}$CD43$^{lo/-}$CD25$^{+}$, B220$^{lo}$IgM$^{+}$, and B220$^{hi}$IgM$^{+}$, respectively. (B and C) Spleen cells were stained with antibodies recognizing B220, IgM, CD21 and CD23 and analyzed by flow cytometry. The numbers of (C) are percentages of cells falling in each gate. Each dot represents one mouse (B). (D) Immunohistological staining of spleen sections with antibodies against IgM (Red) and CD169 (green) and analyzed by confocal flurorescence microscopy. (E) BM chimera mice were generated by reconstituting lethally irradiated recipient mice with BM from donors of B6 (CD45.1) and *Mavs*$^{-/-}$ (CD45.2) at a 1:1 ratio. After 6 weeks of reconstitution, the spleens of recipients were analyzed by flow cytometry as in B and C. Transitional (Tr) B cells were defined as B220$^{+}$AA4.1$^{+}$IgM$^{+}$. Each dot represents a mouse. *p<0.05, *p<0.01, ***p<0.001. ns, not significant.
